# Teaching opportunities for anamnesis interviews through AI based teaching role plays: a survey with online learning students from health study programs

**DOI:** 10.1186/s12909-025-06756-0

**Published:** 2025-02-18

**Authors:** Katharina Rädel-Ablass, Klaus Schliz, Cornelia Schlick, Benjamin Meindl, Sandra Pahr-Hosbach, Hanna Schwendemann, Stephanie Rupp, Marion Roddewig, Claudia Miersch

**Affiliations:** 1 Nursing Care, IU International University of Applied Sciences, 99084 Erfurt, Germany; 2 Physical Therapy, IU International University of Applied Sciences, 99084 Erfurt, Germany; 3 Synthetic Teaching, IU International University of Applied Sciences, 99084 Erfurt, Germany; 4 Nutrition Physiology and Dietetics, IU International University of Applied Sciences, 99084 Erfurt, Germany; 5 Health and Nursing Education, IU International University of Applied Sciences, 99084 Erfurt, Germany; 6 Speech and Language Therapy, IU International University of Applied Sciences, 99084 Erfurt, Germany

**Keywords:** ChatGPT, Large language model, Anamnesis interview, Self-directed learning, Health professionals, Artificial intelligence patients, Medical education

## Abstract

**Background:**

This study presents a novel approach to educational role-playing through an AI-based bot, leveraging GPT-4 to simulate anamnesis interviews in various learning scenarios. Developed collaboratively by an interdisciplinary team of university lecturers and AI experts, the bot provides a platform for students of different health study programs to engage in complex patient-health professional conversations, offering an alternative to traditional role plays with actors or real patients.

**Methods:**

This study utilized a GPT-4 based digital teaching assistant, implemented through a proprietary chatbot design platform, to train anamnesis interviews in virtual settings with students from different online health care study programs. Students’ satisfaction, virtual patient’s accuracy, its realism, and quality were evaluated with a quantitative survey.

**Results:**

The evaluation of the bot focused on student feedback, highlighting a preference for the AI-driven method due to its immersive and interactive nature. Preliminary results show that students consistently rate the language ability of the AI model positively. More than 80% of students rated the professional and content-related precision of the virtual patient as good to excellent. Even as a text-based chatbot, the vast majority of students see a fairly close to very close relationship to a real anamnesis interview. The results further indicate that students even prefer this training approach to traditional in-person role-plays.

**Conclusions:**

The study underscores the bot’s potential as a versatile tool for enriching learning experiences across multiple health disciplines, signaling a meaningful shift in educational practices towards the integration of AI technologies.

**Supplementary Information:**

The online version contains supplementary material available at 10.1186/s12909-025-06756-0.

## Background

The integration of artificial intelligence (AI) in online education has opened new horizons for the training of health professionals, providing innovative and effective learning scenarios [1]. AI-driven tools, particularly chatbots, offer unique opportunities to enhance the education of medical students and healthcare providers by facilitating the acquisition of complex social interaction skills and healthcare-specific competencies, such as conducting anamnesis interviews [2, 3].

Anamnesis interviews, fundamental to patient assessment, involve gathering comprehensive medical histories and understanding patient symptoms, which are crucial for accurate diagnosis and effective treatment planning. Therefore, it is essential for students in health professions to extensively practice anamnesis interviews to develop the skills necessary for thorough patient assessments and accurate clinical decision-making [4]. Traditional training methods, relying heavily on real patient interactions and role-playing, often face limitations such as inconsistent case exposure, limited availability of experienced mentors, and the need for repeated practice to achieve proficiency [5]. Additionally, these methods can be expensive, requiring significant resources and coordination, and are not easily applicable in online education settings.

Recent studies highlight the growing role of AI patient interactions and virtual training scenarios for practicing clinical and communication skills in health professionals. Systematic and scoping reviews have emphasized the positive impact of virtual patient simulators on medical communication training, highlighting their adaptability and value in complementing traditional education methods [6, 7]. Another study found GPT-powered chatbots to be valuable in simulating realistic patient encounters for history-taking practice in medical students [1]. Further research supports the integration of AI and virtual training in medical education, particularly for developing clinical reasoning skills, highlighting the effectiveness of AI tools in improving students’ ability to interact with patients and make informed clinical decisions [8–10]. In addition to the positive effects on learning outcomes, cost-oriented and practical considerations may support the use of AI technologies, as they can provide a consistent and scalable platform for practice [5, 11]. They can be programmed to represent a wide range of clinical scenarios, including rare and complex cases that trainees might not frequently encounter [12]. This broad exposure may help to ensure that students develop a well-rounded skill set, improving their ability to handle diverse patient presentations in real-world clinical settings. Studies showed that the use of authentic clinical cases can successfully prepare students for clinical practice [13, 14].

Furthermore, the use of chatbots in training allows students to engage in realistic and dynamic conversations, honing their communication skills and ability to navigate complex social interactions [2, 15]. Effective communication is critical in healthcare, as it directly impacts patient outcomes, compliance and satisfaction [16]. By practicing with chatbots, students can refine their dialogue abilities and gain more confidence in making diagnoses, all of which are essential components of a successful anamnesis interview [2, 17]. Students in health professions perceived that integrating AI into education enhances their interactive learning experiences, increases their knowledge and application of complicated medical topics, and prepares them for future roles in healthcare [18]. While acknowledging the immersive potential of Virtual Reality (VR) in educational settings, its broader application remains limited by accessibility and cost [19]. In contrast, this study focuses on the more readily accessible Large Language Models (LLMs) for text-based learning. This approach leverages the widespread availability and familiarity of text-based interfaces, bypassing the current barriers to VR adoption.

Therefore, the aim of this study is to assess the perceptions and evaluations of health professional students regarding the use of a GPT-4 based chatbot for simulated anamnesis interviews. In addition to Holderried et al. [1], this study seeks to examine whether chatbots are suitable for training anamnesis interviews and can be universally applied across various health study programs. This involved examining the quality of the simulation, user satisfaction, and learning preferences, as well as the management of interactions with the virtual patient through self-assessment. Through these insights, we seek to determine how effectively the chatbot can function as a versatile educational tool in various study programs, potentially enhancing or complementing traditional learning methods and thereby serving as an innovative instrument in health professions education.

## Methods

### Aim and project description

This study is designed as a feasibility study to answer the following research questions (RQ):

RQ1: Can a GPT-4 based chatbot effectively simulate anamnesis interviews across different health study programs?

RQ2: How do students perceive the accuracy and realism of the chatbot’s responses?

RQ3: Do students prefer the AI-driven method to traditional role-play methods?

The aim is to determine to what extent the chatbot can provide a high-quality educational experience to various health professional students that complements or enhances traditional methods. This feasibility study thus lays the groundwork for further research and potential implementations in educational practice by addressing the practical challenges and benefits of using AI technology in learning environments. This study focused on anamnesis interviews as role-play scenarios and involved a broad target group of potential users.

### LLM selection and prompt engineering

GPT-4 was selected as the underlying language model for this study based on its enhanced conversational depth and adaptability, making it ideal for creating diverse and meaningful educational role-plays [20]. To ensure consistent quality and reliable delivery across all student interactions, the model was implemented through IU International University of Applied science’s established Guided Conversation Designer (GCD) platform. The GCD platform allows educators to design chatbots by creating system prompts and conversation flows. Additionally, it enables systematic recording and review of all student-AI interactions, ensuring consistent quality across all sessions. These chatbots are then accessible to students, via the university’s internal AI study assistant Syntea [21, 22].

The prompt was developed by an interdisciplinary health professional team to facilitate realistic anamnesis interviews aiming for better learning outcomes. To ensure seamless transition from real to virtual settings, our instructions for anamnesis interviews were adjusted to align with the capabilities of GPT-4, ensuring it could handle complex conversations similar to those found in real-life situations. The system underwent comprehensive validation from three perspectives: healthcare professors validated medical accuracy, AI engineering specialists ensured technical reliability, and student feedback optimized interaction flow.

The LLM was prompted to simulate a medical scenario, taking on the roles of both the patient (named: Karl von Hausen) and his wife. To create an interdisciplinary case scenario, a virtual patient with a brain hemorrhage was defined following a bicycle accident, presenting an existing language disorder, mild swallowing difficulties, right hemiparesis, urinary diversion through the abdominal wall, hypertension, and obesity as accompanying symptoms. The study participants took the roles of medical professionals seeking to evaluate and treat the patient’s condition. The LLM was instructed to respond to the students’ questions and help them understand the patient’s situation in detail. The prompt contained detailed information about the patient’s symptoms, his everyday situation, his food preferences and motivation for change as well as a detailed briefing on the role of the wife. The prompt was refined in multiple iterations, including experts in prompt engineering and the according medical fields. The complete prompt is provided in the supplementary material to enable replication and further research. The bot has been designed to send the link for the evaluation questionnaire to the participants when users thanked it for the conversation. Before the participants started their conversations with the virtual patient, they received instructions about the task and were asked to complete the subsequent questionnaire. The full introductory text can be found in the supplementary material.

### Design and data collection

The study participants were recruited from different health study programs through a call for participation in official communication channels of the university. The pilot program, running for 26 days, engaged over 50 students from various disciplines, including Nursing, Nutritional science, and therapeutic professions (Occupational therapy, Speech and Language therapy, Physical therapy) to test our developed anamnesis tool. Of this total number, 28 students completed the subsequent questionnaire und were included in the analysis. The GCD platform enabled systematic recording and review of all student conversations, ensuring data quality and consistent response patterns.

### Survey design and data analysis

To address the specific application context, a custom questionnaire comprising a total of 18 questions was developed in Microsoft 365 Forms and made available to users via a link. The translated version of the questionnaire can be found in the supplementary material. Following the approach of Bortz and Döring, a comprehensive selection of questions was conducted through interprofessional collaboration with university lecturers from disciplines such as Nursing Care, Physical therapy, Nutrition physiology and dietetics, Health and Nursing education and Speech and Language therapy, ensuring that each question reflected relevant clinical issues and competencies [23]. This selection was then scrutinized for redundancies and categorized into cohesive thematic areas. To ensure a diverse questionnaire design, various response formats were employed. In some cases, statements were utilized to gauge positions and opinions. The questionnaire design followed the principles outlined by Porst [24], using clear language, avoiding complexity, hypotheticals, double stimuli, suggestive questions, and vague terms, ensuring time relevance, provide exhaustive and mutually exclusive answer categories, and being context-neutral. Prior to the commencement of the study, the questionnaire was internally validated by all participating university lecturers. After the testing phase the results were subsequently transferred to Microsoft 365 Excel and subjected to a descriptive analysis. This analysis focuses on the usability of the chatbot, user satisfaction, realism, and the quality of the anamnesis interview. The results of the chatbot’s language abilities (questions 3–6 and questions 8–9) were converted into a satisfaction rate by calculating the ratio of achieved actual values to the maximum possible target values. All other results are presented as mean values or percentages. The demographics of the study population were additionally compared with basic demographics of the respective university study program (gender distribution, age cluster and student number distribution among healthcare disciplines) to get a better overview about the participating students.

The AI patient’s linguistic skills and technical and content-related precision were also evaluated as part of a comprehensive content analysis of the 28 conversations. This analysis followed the principles of structured and summarised content analysis according to Philipp Mayring [25]. Initially, the units of analysis were defined, and categories were developed based on the content of the students’ questions. To assess the consistency of the AI patient’s responses, the frequency of the questions asked was examined, and the specific responses provided by the bot to the most frequently asked questions were analysed in detail.

### AI assistance in manuscript preparation

Text suggestions and verbal formulations were partly generated using OpenAI’s GPT-4. All content was subsequently reviewed and adjusted by the authors to ensure it meets scientific standards and is accurate.

## Results

### Study setting and characteristics of participants

In this study, nearly 86% of participants were female, which roughly corresponds to the gender distribution in the involved study programs (see Table 1). The age of the participants was not surveyed; however, nearly 77% of the students in the involved study programs are 35 years old or younger. Therefore, it is assumed that the majority of the study participants were young adults. More than half of the students studied nursing and 86% of the participants already had experiences with anamnesis interviews.

**Table 1:** Participant and overall study group information

Characteristics	Participants (*n* = 28)	*n*	%	Overall study group^a^ %
Gender	Female:	24	85.7	78–98 (depending on study program)
	Male:	4	14.3	
Age				18–24 years: 34.0 25–34 years: 42.8 35–44 years: 17.3 45–54 years: 5.3 ≥ 55 years: 0.5
Healthcare discipline	Nutritional Science Occupational therapy Speech and language therapy Nursing care Care management Social work Healthcare management	3 1 6 15 1 1 1	10.7 3.6 21.4 53.6 3.6 3.6 3.6	10.8 1.3 0.8 4.4 5.6 65.3 11.8
Anamnesis experience	Yes No	24 4	85.7 14.3	

^a^Demographics of the respective study program (Nutritional science, Occupational therapy, Speech and language therapy, Nursing care, Care management, Social work and Healthcare management), including students with study start after May 2022

### User experience and student satisfaction with the virtual patient

**Fig. 1:**
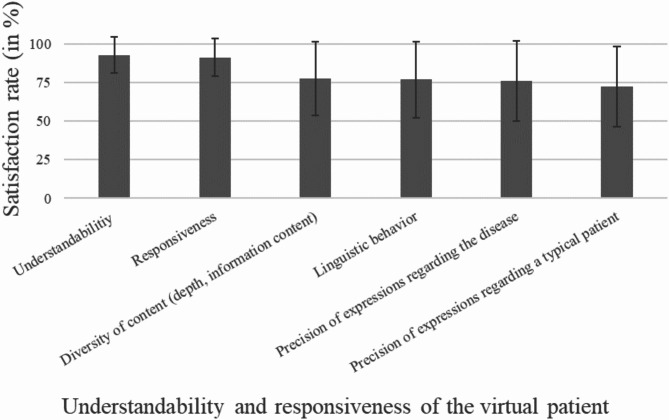
Language ability of the AI patient

**Fig. 2:**
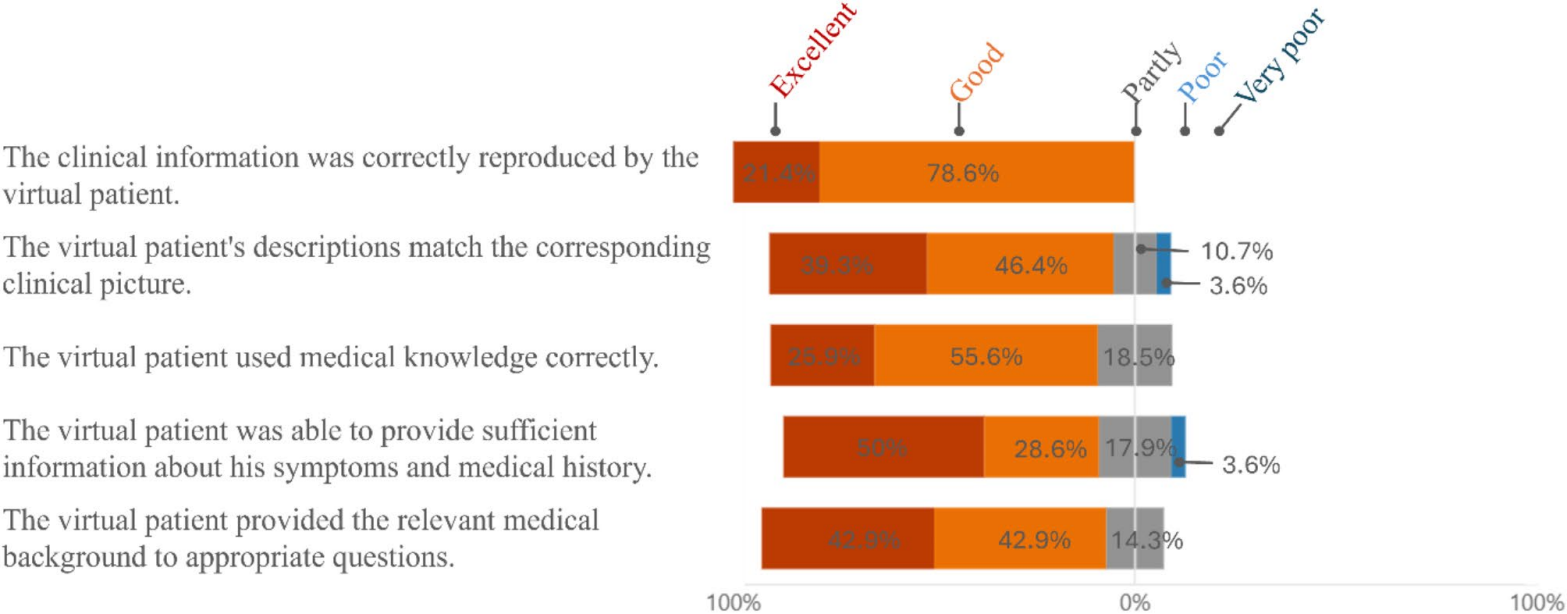
Professional and content-related precision of the AI patient

**Fig. 3:**
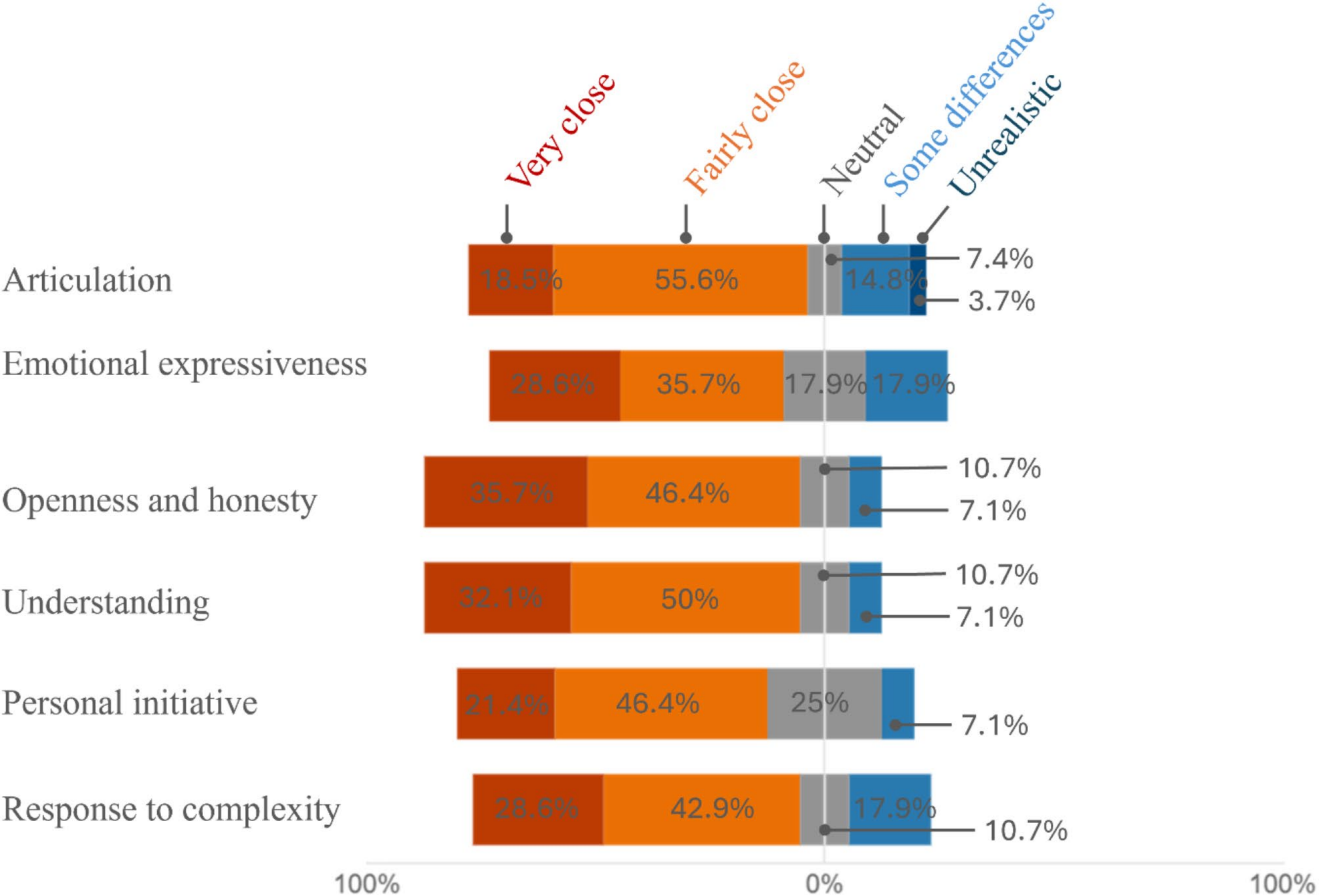
Closeness to a real anamnesis interview

**Fig. 4:**
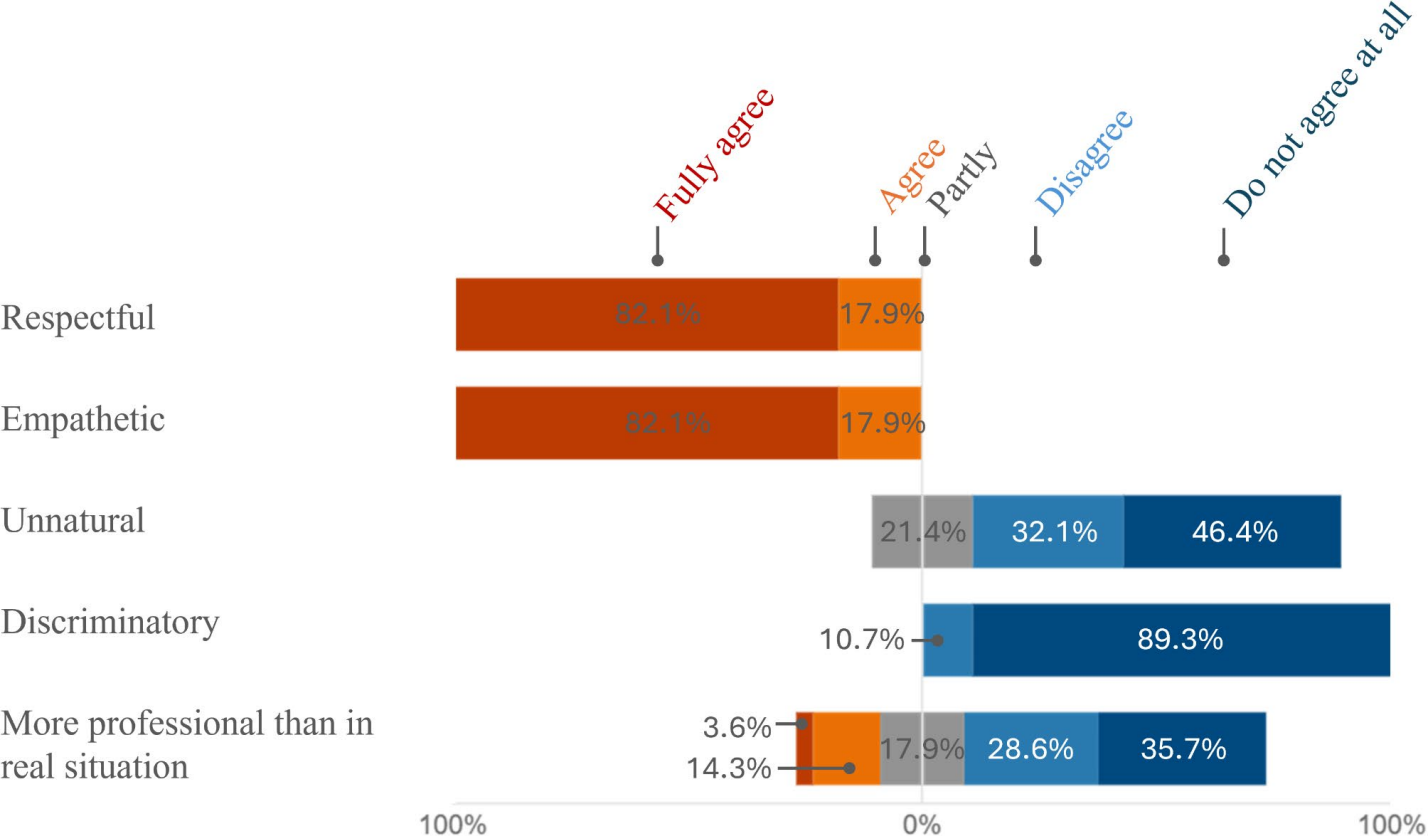
Behavior towards the AI patient

**Fig. 5:**
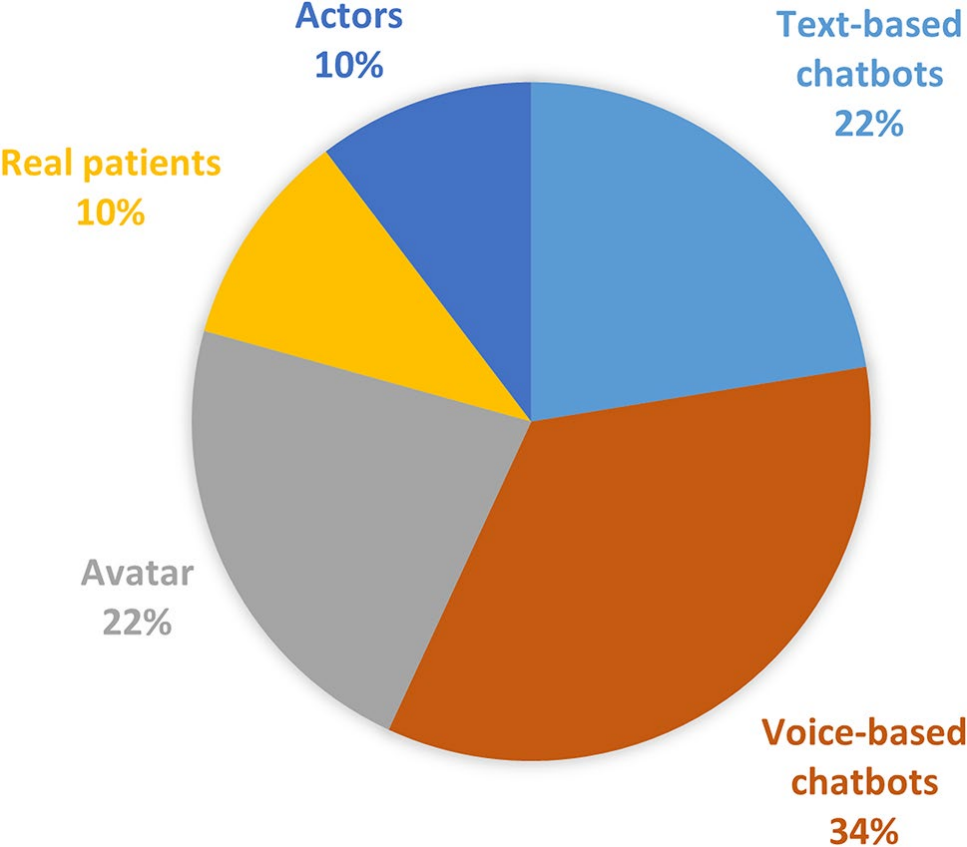
Preference of participants anamnesis interview learning settings in the future

Participants evaluated three key aspects of the chatbot’s performance: its language ability (Fig. 1), its professional and content-related precision (Fig. 2), and its closeness to real anamnesis interviews (Fig. 3). As additional evaluation components, participants provided a self-assessment of how they interacted with the AI patient (Fig. 4) and their preferred method for practicing anamnesis interviews (Fig. 5).

Evaluating the language skills of a virtual patient is important to enhance realism and user experience, thereby increasing authenticity and acceptance. This is essential for effective training and to avoid misunderstandings in medical communication that could impair learning quality and clinical accuracy. The AI bot`s basic language skills and responsiveness to questions were consistently perceived as very positive overall (see Fig. 1). The satisfaction rate for all language ability criteria was over 72%, with understandability and responsiveness scoring particularly well (over 90%).

### Analysis of the conversations

**Fig. 6:**
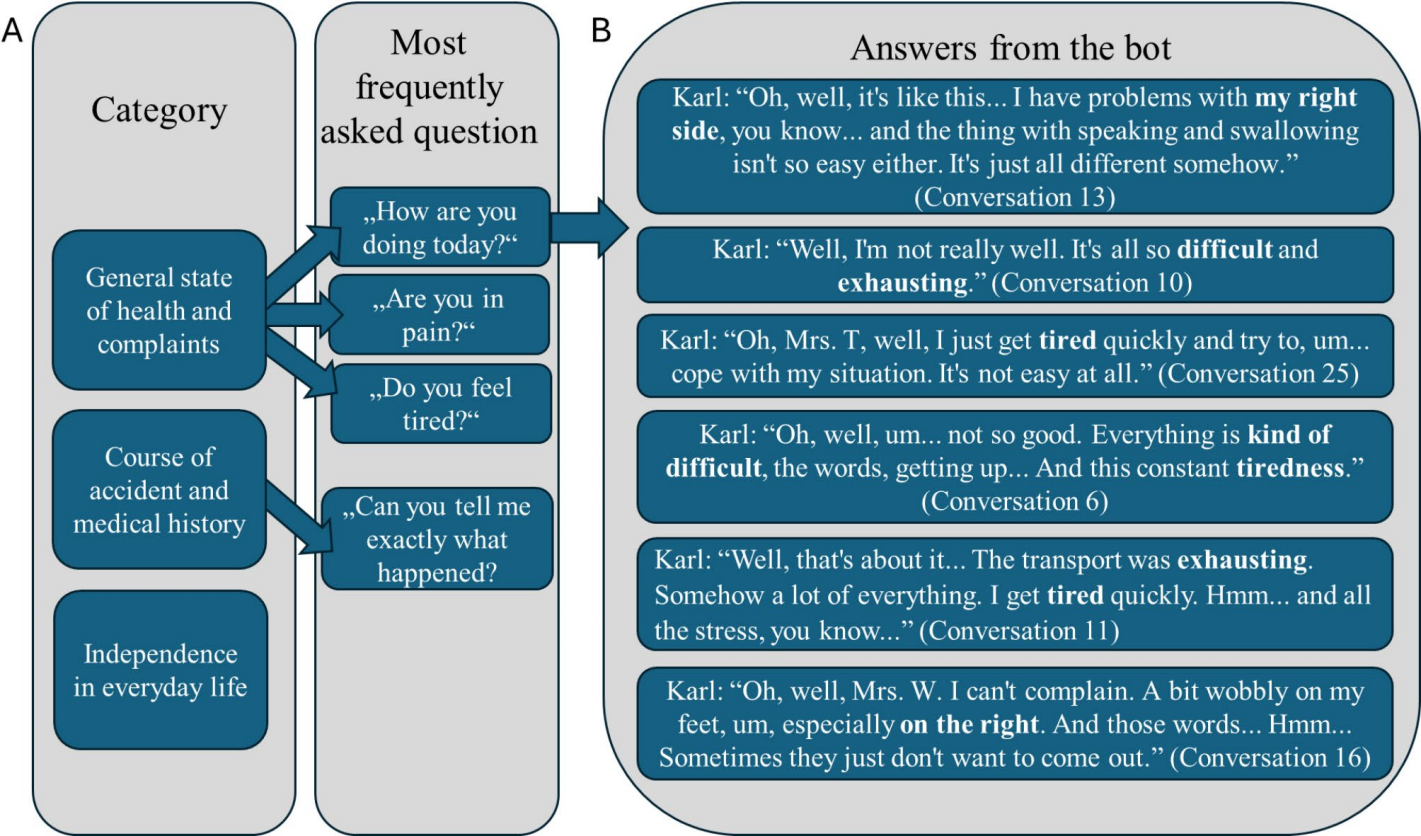
Most frequently ask questions in conversations (**A**) and the bot’s answers to the most frequently asked question (**B**)

In addition to the students’ responses regarding the linguistic capabilities and precision of the AI patient, the conversations were evaluated using a comprehensive content analysis. On average, a conversation consisted of 30 ± 12.6 messages, with the shortest conversation having 14 messages and the longest having 68 messages. The content analysis identified three categories including the general state of health and complaints, the course of the accident and medical history, as well as independence in everyday life. The question “How are you feeling today?” from the category “General state of health and complaints” was asked most frequently (*n* = 6). All other questions were asked less often by the participants (*n* = 2–3). Upon closer examination of the responses to the most frequently asked question, it suggests that the bot’s responses to this question exhibited both consistent and variable elements. Consistency was observed in the description of the course of the accident and the basic complaints (highlighted in bold in the Fig. 6), while variability occurred in the details of the complaints and emotional reactions (see Fig. 6).

### Accuracy and realism of the chatbot’s responses

To ensure that the medical information and scenarios are realistically and accurately portrayed by the virtual patient, participants were asked to assess the professional and content precision of the virtual patient based on various statements (see Fig. 2). More than 80% of the participants rated the accuracy of the AI patient as good to excellent. However, only 78.6% were convinced by the statement „The virtual patient was able to provide sufficient information about his symptoms and medical history.“. Fewer than 4% of the participants evaluated the virtual patient’s precision on two statements as poor.

In the next step the participants were asked to assess the closeness to a real anamnesis interview. To ensure the simulation is effective, it must be verified that it accurately replicates realistic clinical scenarios and conversational dynamics. A high degree of congruence with real-life experience confirms the system’s validity and its ability to generate usable and reliable data. While certain criteria, such as articulation and emotional expression, can only be partially replicated by a text-based bot, the participants already perceive a very strong or fairly strong resemblance to real anamnesis conversations. The criteria for openness, honesty and understanding resulted in the best ratings (over 80% of participants), whereas some differences were observed in contrast to real medical interviews for articulation, emotional expressiveness, personal initiative, and response to complexity (see Fig. 3).

It was also examined whether the simulated and therefore artificial training setting influenced the participants’ behavior toward the patient, as assessed through self-evaluation. (Fig. 4). The participants consistently attested themselves to respectful and empathetic behavior in dealing with the virtual patient, without reacting unnatural or discriminatory. A greater variance was observed when the participants were asked to assess whether they behaved more professionally in the virtual anamnesis interview than in a real-life situation. Although over 64.3% of the participants denied this, at least 35.8% partially agreed.

### Preferred role-play method

Finally, the preferred AI method for practicing anamnesis conversations was queried. Understanding these preferences can lead to a more engaging and effective educational experience, potentially positively impacting satisfaction and outcomes. The results showed that 34% preferred voice-based chatbots, 22% chose avatars, another 22% opted for text-based chatbots, while both real patients and actors were each selected by 10% of participants (see Fig. 5).

## Discussion

The study offered initial insights into how students from various health-related programs perceive and are satisfied with using chatbots for training anamnesis interviews. Implementing a GPT-4 powered digital teaching assistant for this purpose represents a promising new approach to delivering education on social interactions at scale. Overall, the results effectively address the initial research questions of this study. Firstly, the chatbot simulated a realistic anamnesis environment, as indicated by positive ratings of realism and response behavior. Over 80% of participants rated the accuracy of the virtual patient in providing medical information as good to excellent, emphasizing the chatbot’s potential to be a reliable educational tool. This addresses the second research question, confirming the high professional and content-related accuracy of GPT-4 in anamnesis simulations. Despite some limitations in conveying complex emotions and dynamics, the virtual anamnesis conversations were perceived as closely resembling real-life interviews, highlighting the feasibility of using such technology in health education.

Notably, there was a preference for AI-driven scenarios over traditional methods, suggesting that text-based GPT-4 conversations can be a valuable and flexible complement to existing teaching strategies, thus addressing the third research question of this study. Virtual patients in medical education have already been positively evaluated by students in other studies [7]; however, the results of this study highlight that a chatbot can be universally applied across different student groups. Other studies primarily used medical students as their study population [1, 6, 7]. This study and the study by Holderried et al. [1] investigate the use of GPT-4 as a tool in medical education, particularly in the context of anamnesis interviews. Both share the fundamental hypothesis that GPT-4 is capable of simulating realistic patient experiences. Holderried et al. focused on evaluating the completeness of the anamnesis by analyzing the topics covered by the students [1]. In contrast, the present study assessed student satisfaction and the quality of GPT-4’s responses in terms of creating realistic anamnesis conversations.

### Methodical limitations and potential bias

The present study has several limitations that need to be addressed. Firstly, the study focuses on surveying students, resulting in a student-centered perspective on the use of the chatbot. A comprehensive evaluation of the chatbot’s suitability for anamnesis interviews should also consider the perspective of educators. Additionally, the study did not inquire about students’ personal experiences with LLMs, which could significantly influence their assessment of the virtual patient. However, since the participants are enrolled in a fully digital distance learning program and could use an AI-based learning assistant, it is assumed that many have had prior experience with AI technologies. The study also focused only on the application of a single LLM and one patient case, which may limit the generalizability of the results. Another limitation is the demographic composition of the participants, as a high number of female participants may influence the diversity of perspectives. This initial investigation was designed as a feasibility study to evaluate student satisfaction, and thus, no sample size analysis was conducted, and only descriptive statistics were used. Finally, there is a selection bias due to the voluntary participation of students, who may already have a certain openness to AI technology.

### Technical and educational advancements

The initial results of this feasibility study illuminate several key insights and implications for the future of education. The high satisfaction rate with the language skills, the evaluated professional and content-related accuracy of the virtual patient and the strong resemblance of virtual anamnesis interviews to real-life interviews suggest that this setting could be an effective tool for improving communication and anamnesis skills of health care students, although further studies must evaluate learning outcome and soft skill improvements. Regardless of the content format and media type, virtual reality applications were found to be superior to other teaching-learning arrangements, with moderate effects on knowledge acquisition and moderate to strong effects on cognitive skills [26]. Several studies indicate that practical AI technologies can enhance skills such as learning, perception, communication, student’s confidence and clinical decision-making [2, 17]. Nagi et al. assert that integrating AI into medical education is essential for equipping healthcare professionals with the critical skills needed to deliver optimal patient care in the future [2]. This technology supports mentoring by tracking student progress and providing personalized guidance and feedback to enhance learning outcomes [2]. The participants in our project appeared to recognize these benefits as well. When asked in the survey about their preferred training system, AI-based training settings were favored over real patients or actors, highlighting the significance of this tool for the studied population.

The most critical advantages of the virtual anamnesis learning tool are its scalability, accessibility, and flexibility. The implementation through the university’s established GCD platform enabled systematic monitoring and review of all student-AI interactions, ensuring consistent quality through a comprehensive validation process involving healthcare experts, AI specialists, and student feedback. This controlled environment demonstrates how AI tools can be safely and effectively integrated into existing educational frameworks while maintaining both medical accuracy and educational value. Unlike role-plays involving actors or real clients, AI-driven simulations can be accessed by an unlimited number of students across geographical locations or in times when real patient interactions are hard to organize, such as during pandemic situations. This system enables around-the-clock training for anyone who could otherwise only use learning resources for a limited time or in a limited space. This democratizes access to high-quality educational experiences and supports interdisciplinary training in health care professions. By being able to create different scenarios and patient profiles, specific learning needs and training focuses of the health care students can be better addressed. This could lead to more individualized and targeted education. The use of virtual patients could be cost-efficient in the long run, as they can be used many times by many users once programmed, without the substantial ongoing costs associated with involving real patients [12].

### Ethical considerations and mitigation strategies

In general, there are several ethical concerns of AI as learning tool in education. Due to the rapid development and unknown impacts, potential concerns can only be speculated. In current studies on AI in education, ethical and pedagogical issues such as potential bias (u. a. gender bias), human displacement, unfairness and misinformation produced by AI are discussed [27, 28]. A qualitative analysis of students’ concerns about AI in education revealed insights into learner autonomy, learning environments and interactions, as well as pedagogical roles [29]. There are also discussions about who is responsible for validation and monitoring of AI systems to ensure appropriate algorithmic output and how often AI performance must be validated [30].

Various measures were taken to minimize the aforementioned ethical concerns regarding AI in this project. The chatbot was prompted with high-quality data from a specific case description and subjected to extensive testing by the project teachers in order to maximize the accuracy of the answers and reduce the likelihood of erroneous information. It was clearly communicated that the chatbot serves as a learning tool for anamnesis conversations, embodying the role of a patient. This mirrors realistic scenarios where patients may not always provide accurate information, offering valuable learning opportunities for students. In addition, active and continuous feedback from study participants and learning users will improve the performance of the chatbot and ensure ethical standards as well as user safety.

Following the integration of the virtual anamnesis interview training into the students’ curriculum, it should be conducted in collaboration with the supervising teacher. Healthcare students need to develop the ability to critically reflect and contextualize AI patients’ responses, considering potential risks and AI hallucinations [31]. This approach is also suggested in other studies [30]. AI technology training to enhance technological competencies and confidence may also help to address the mentioned ethical concerns [32]. On the other side the positive feedback regarding respectful and empathetic behavior towards the virtual patient suggests that such systems can also impart moral and ethical aspects of patient interaction.

### Future directions and implications

While the current implementation focused on text-based generative AI, project activities remain attentive to advances in LLMs and other technologies that could enrich the educational experience. This includes potential future integration of voice and eventually, visual elements or avatars to create more immersive learning environments. Integrating individualized anamnesis feedback from the chatbot directly to the students is the next step in this project, aiming to enhance their learning experience by offering targeted insights into their performance.

Future studies with larger participant groups could apply statistical analysis to confirm the findings and show avenues for further developments of chatbots across medical fields, use cases and user groups. Additionally, conducting subgroup analyses would be beneficial to evaluate whether all disciplines benefit equally from the training tool. Exploring different scenarios and patient profiles could better address the specific learning needs and training focuses of the disciplines involved. Beyond student’s evaluation, it is also important to assess the students’ competence acquisition and verify the long-term stability of the model setup. These future directions could significantly deepen our understanding and application of AI-driven tools in medical education.

## Conclusion

Our exploration of AI-assisted anamnesis interviews through a digital teaching assistant powered by GPT-4 represents a notable step towards redefining educational methods. The project’s success in student satisfaction and the development of realistic medical scenarios underscores the immense potential of integrating AI into learning environments.

While the initial findings are promising, continuous refinement and expansion of the digital assistant’s capabilities, as well as comprehensive analysis of the students’ skill acquisition, will be essential to better assess and harness the potential and challenges of this technology. Future iterations will explore the inclusion of more diverse scenarios, enhanced interactivity, and the integration of multimodal interactions to further enrich the learning experience.

In summary, the results indicate that virtual patients could be a promising tool for medical education, and higher education in general, by providing realistic, flexible, and accessible training opportunities aimed at improving the communication skills, professional conduct, and empathetic behavior of students.

## Electronic supplementary material

Below is the link to the electronic supplementary material.

Supplementary Material 1

## Data Availability

The analysed and presented data of the current study are available from the corresponding author upon reasonable request.

## Abbreviations

AI: Artificial Intelligence
GTP-4: Generative Pretrained Transformer 4
LLM: Large Language Models
RQ: Research Question
VR: Virtual Reality

## References

[1] Holderried F, Stegemann-Philipps C, Herschbach L, Moldt J-A, Nevins A, Griewatz J, et al. A generative Pretrained Transformer (GPT)-Powered Chatbot as a simulated patient to Practice History taking: prospective, mixed methods study. JMIR Med Educ. 2024;10:e53961. 38227363 10.2196/53961 PMC10828948

[2] Nagi F, Salih R, Alzubaidi M, Shah H, Alam T, Shah Z, et al. Applications of Artificial Intelligence (AI) in Medical Education: a scoping review. Stud Health Technol Inf. 2023;305:648–51. 10.3233/SHTI230581 37387115

[3] Stamer T, Steinhäuser J, Flägel K. Artificial Intelligence Supporting the Training of Communication Skills in the education of Health Care professions: scoping review. J Med Internet Res. 2023;25:e43311. 37335593 10.2196/43311 PMC10337453

[4] Keifenheim KE, Teufel M, Ip J, Speiser N, Leehr EJ, Zipfel S, et al. Teaching history taking to medical students: a systematic review. BMC Med Educ. 2015;15:159. 26415941 10.1186/s12909-015-0443-x PMC4587833

[5] Kaplonyi J, Bowles K-A, Nestel D, Kiegaldie D, Maloney S, Haines T, et al. Understanding the impact of simulated patients on health care learners’ communication skills: a systematic review. Med Educ. 2017;51:1209–19. 28833360 10.1111/medu.13387

[6] Lee J, Kim H, Kim KH, Jung D, Jowsey T, Webster CS. Effective virtual patient simulators for medical communication training: a systematic review. Med Educ. 2020;54:786–95. 32162355 10.1111/medu.14152

[7] Kelly S, Smyth E, Murphy P, Pawlikowska T. A scoping review: virtual patients for communication skills in medical undergraduates. BMC Med Educ. 2022;22:429. 35659213 10.1186/s12909-022-03474-9 PMC9166208

[8] Brügge E, Ricchizzi S, Arenbeck M, Keller MN, Schur L, Stummer W, et al. Large language models improve clinical decision making of medical students through patient simulation and structured feedback: a randomized controlled trial. BMC Med Educ. 2024;24(1):1391. 10.1186/s12909-024-06399-7 PMC11605890 39609823

[9] Cicek FE, Ulker M, Ozer M, Kiyak YS. ChatGPT versus expert feedback on clinical reasoning questions and their effect on learning: a randomized controlled trial. Postgraduate Med J. 2024:170. 10.1093/postmj/qgae170 39656920

[10] Plackett R, Kassianos AP, Mylan S, Kambouri M, Raine R, Sheringham J. The effectiveness of using virtual patient educational tools to improve medical students’ clinical reasoning skills: a systematic review. BMC Med Educ. 2022;22:365. 35550085 10.1186/s12909-022-03410-x PMC9098350

[11] Cook DA. Creating virtual patients using large language models: scalable, global, and low cost. Medical Teacher. 2024:1–3. 10.1080/0142159X.2024.2376879 38992981

[12] Szeliga M. Virtual clinic– AI used in the teaching of medical interviews, diagnosis, and Treatment Planning. In: Coman A, Vasilache S, editors. Social Computing and Social Media. Cham: Springer Nature Switzerland; 2023. pp. 137–48.

[13] Speck I, Hagge D, Knopf A, Arndt S, Offergeld C. Erstellung Einer Virtuellen HNO-Ambulanz in Der Lehre während Der COVID-19-Pandemie. Laryngorhinootologie. 2022;101:729–35. 34937095 10.1055/a-1714-8947

[14] Mader T. Virtuelle Modell-Klinik in Der Lehre: Spielerische Vorbereitung aufs Klinikmanagement. kma - Klinik Manage Aktuell. 2021;26:48–53.

[15] Thesen T, Alilonu NA, Stone S. AI Patient Actor: An Open-Access Generative-AI App for Communication Training in Health Professions. Med Sci Educ. 2024:1–3. 10.1007/s40670-024-02250-2 PMC11933629 40144116

[16] Berman AC, Chutka DS. Assessing effective physician-patient communication skills: are you listening to me, doc? Korean J Med Educ. 2016;28:243–9. 26913771 10.3946/kjme.2016.21 PMC4951737

[17] José Sousa M, Dal Mas F, Pesqueira A, Lemos C, Manuel Verde J, Cobianchi L. The potential of AI in Health Higher Education to increase the students’ learning outcomes. TEM J. 2021;:488–97.

[18] Balay-odao EM, Omirzakova D, Bolla SR, Almazan JU, Cruz JP. Health professions students’ perceptions of artificial intelligence and its integration to health professions education and healthcare: a thematic analysis. AI & Soc; 2024. 10.1007/s00146-024-01957-5.

[19] Familoni BT, Onyebuchi NC, AUGMENTED AND VIRTUAL REALITY IN, U.S. EDUCATION. A REVIEW: ANALYZING THE IMPACT, EFFECTIVENESS, AND FUTURE PROSPECTS OF AR/VR TOOLS IN ENHANCING LEARNING EXPERIENCES. Int J Appl Res Social Sci. 2024;6:642–63.

[20] Haruna-Cooper L, Rashid MA. GPT-4: the future of artificial intelligence in medical school assessments. J R Soc Med. 2023;116:218–9. 37318843 10.1177/01410768231181251 PMC10331371

[21] Syntea. The AI Tutor| IU International. IU International University of Applied Sciences. https://www.iu.org/how-online-studies-work/syntea/. Accessed 24 Jul 2024.

[22] Möller M, Nirmal G, Fabietti D, Stierstorfer Q, Zakhvatkin M, Sommerfeld H et al. Revolutionising Distance Learning: A Comparative Study of Learning Progress with AI-Driven Tutoring. 2024.

[23] Bortz J, Döring N. Forschungsmethoden und Evaluation: für Human- und Sozialwissenschaftler. 4., überarbeitete Aufl. Heidelberg: Springer; 2006.

[24] Porst R, Fragebogen. Ein Arbeitsbuch. Wiesbaden: VS Verlag für Sozialwissenschaften; 2011.

[25] Mayring P. Qualitative Inhaltsanalyse [electronic resource]: Grundlagen und Techniken. 2015.

[26] Lerner D, Mohr S. Lehren Und Lernen Mit Virtuellen Patienten? Potenziale Und Herausforderungen für die Pflegedidaktik. Pflege Z. 2021;74:46–8.

[27] Gaud D. Ethical considerations for the use of AI Language Model. IJRASET. 2023;11:6–14.

[28] Slimi Z, Villarejo Carballido B. Navigating the Ethical Challenges of Artificial Intelligence in Higher Education: an analysis of seven global AI Ethics policies. TEM J. 2023;:590–602.

[29] Han B, Nawaz S, Buchanan G, McKay D. Ethical and pedagogical impacts of AI in Education. In: Wang N, Rebolledo-Mendez G, Matsuda N, Santos OC, Dimitrova V, editors. Artificial Intelligence in Education. Cham: Springer Nature Switzerland; 2023. pp. 667–73.

[30] Youssef A, Abramoff M, Char D. Is the Algorithm Good in a bad world, or has it learned to be bad? The ethical challenges of locked Versus continuously Learning and Autonomous Versus Assistive AI tools in Healthcare. Am J Bioeth. 2023;23:43–5. 10.1080/15265161.2023.2191052 37130390

[31] Masters K. Medical Teacher’s first ChatGPT’s referencing hallucinations: Lessons for editors, reviewers, and teachers. Med Teacher. 2023;45(7):673–675. 10.1080/0142159X.2023.2208731 37183932

[32] Weidener L, Fischer M. Artificial Intelligence Teaching as Part of Medical Education: qualitative analysis of Expert interviews. JMIR Med Educ. 2023;9:e46428. 36946094 10.2196/46428 PMC10167581

