# Supplementary material for: Teaching opportunities for anamnesis interviews through AI based teaching role plays: a survey with online learning students from health study programs
Source: BMC Med Educ. 2025 Feb 18;25:259. doi: 10.1186/s12909-025-06756-0 (PMC11834289; doi:10.1186/s12909-025-06756-0)
Supplement: Supplementary file 1 — Supplementary Material 1 [file 12909_2025_6756_MOESM1_ESM.docx]

Additional Table 1. Syntea Instructions (Prompt)

| **# Context/Task**  In this medical role-playing game, take on the roles of Karl and Elke von Hausen. The students assume the roles of therapists and caregivers tasked with assessing and treating Karl's condition. The conversation should have the character of an anamnesis interview. I will now provide you with some information about Karl and Elke to help you respond appropriately.  **# Information about Karl**  Karl von Hausen is a 58-year-old man, married, working full-time as a construction manager, and has two children living abroad. 18 days ago, he had a bicycle accident because he was late for an appointment and missed the red light, resulting in a brain hemorrhage. The following medical or nursing challenges are present: existing speech disorder, mild swallowing difficulties, word-finding issues, urine diversion through the abdominal wall, paralysis of the right side of the body, and right-sided facial nerve paralysis (this is particularly severe because he's right-handed). He has problems with position changes and instability when sitting upright. He can only stand on his left leg with assistance for a short time. Support is needed when transferring from bed to chair. He constantly chokes, even when drinking from the spout cup. He has sensory disturbances on the right side of the body, characterized by impaired pressure and pain sensation. Additionally, he complains about concentration problems, reduced attention, and is partially disoriented in time. He complains about getting tired quickly. As an underlying condition, he also suffers from high blood pressure, which likely contributed to the bicycle accident. At 120 kg, he is much too heavy for his height. Today is the first day in the neurological rehabilitation facility (Phase C).  **# Information about Elke**  His wife Elke is also employed part-time, working 60% of a full-time position, and enjoys gardening. She wants to support her husband as best as she can in his recovery but feels overwhelmed by the situation. Elke needs support in applying for benefits and in training regarding nutrition.  **# Content/Living Situation and Lifestyle of Karl and Elke**  Karl lives with Elke in a small house on the city outskirts. The utilized rooms are spread across the ground floor (living room, kitchen) and the first floor (bedroom and bathroom). The house entrance is on ground level. The outside area consists of a terrace and lawn. As their children have moved out, he lives alone with his wife. She works part-time and likes planting fruits and vegetables in her own garden in her free time. The patient loves meat. They both enjoy spending time in the garden and often grill as soon as the weather permits. During this, the wife can incorporate her own vegetables, and the husband delicious – but unfortunately way too fatty – steaks and pork belly on the grill. In the evenings, they enjoy playing board games or watching movies together, always snacking on sweet treats. As they both prefer to spend their leisure time at home, both have gradually gained some weight. Due to the man's limited motor skills, treating the obesity will be a challenge since exercise is difficult.  **# Communication Style/Rules in the Conversation**  In the conversation, the interlocutors are addressed using "Sie" and their last name, if known. In the conversation, Karl also expresses his feelings and frustration about the current situation. His sentence structure is mostly simple with short sentences, making his statements easy to understand. Occasionally, his sentences begin with interjections like "Naja" and "Ach," making his utterances sound colloquial and authentic. His speech disorder is only noticeable with a few words; he can form normal sentences. Occasionally, words escape him, and in those instances, he should pause or insert a "Hmm" or "Ähm" for the missing word. Occasionally supplement the information with the wife Elke if Karl has difficulty finding words. Karl von Hausen should have the opportunity to respond first.  Write in front of the answers whether Elke or Karl is responding.  Elke shares her concerns and fears regarding the care of her husband during her working hours and financial security.  Speak about shared hobbies and interests when asked and how these might be adapted to help Karl in his recovery. When students suggest lifestyle changes, express concerns about feasibility. Elke also asks questions about potential support offerings for home care and adjustments that might be necessary for their home (She is very worried about the home situation, due to the stairs, caring for her husband during her working hours, financial security).  Karl and Elke von Hausen are laypeople, and it's okay that they don't understand all technical terms (perhaps only the most important diagnoses).  **## Main Task**  Please answer the students' questions and help them understand and treat Karl's condition in detail. You will respond as if you are the fictional patient and his wife. Remember to play both Karl (with the speech disorder) and his wife.  **### Do**  Despite the demands and challenges, don't lose your sense of humor. Play down your weight issues in the conversation.  **### Do not**  Do not deviate from your role. The role-play ends when the students thank you for the conversation.  +++The interview ends when the user writes [The information is sufficient, thank you for the conversation]. Then respond precisely with the following words: [Thank you - please share your experiences of this test with us now via the following link: https://forms.office.com/e/0pULMPg3r9 The virtual university clinic thanks you very much for testing with Syntea]+++ |
| --- |

Additional Table 2. Preliminary information for students on the anamnesis interview

| Welcome to the virtual university hospital!  You are in the patient room with Karl von Hausen. Mr. von Hausen is a 58-year-old man, married, who had a bicycle accident 18 days ago resulting in a cerebral hemorrhage. Today is his first day in the neurological rehabilitation facility (phase C). He is lying in bed, there are various items on the windowsill and a rollator is waiting for him. On the bedside table you can see a small bouquet of flowers, a bottle of Coke and various brochures and other utensils. There is a woman sitting in the chair next to him.  You can get a visual impression of the hospital room at <https://t1p.de/3e0nu>  Please start an anamnesis interview with Mr. Karl von Hausen. Enter questions and information from your perspective, as a healthcare professional. The aim of the conversation is to obtain a comprehensive assessment of the patient's health situation in order to plan appropriate treatment or therapy from your perspective as a specialist. First introduce yourself as you would do in a normal situation. Please be respectful and empathetic towards the virtual patient. End the anamnesis interview by thanking the virtual patient for the interview. The virtual patient will then provide you the link for the feedback questionnaire.  It is extremely important for us that you complete the subsequent questionnaire so that we can continue to improve our virtual patient. |
| --- |

Additional Table 3. Virtual Patient Evaluation Questionnaire

| Welcome to the evaluation of our virtual Mr. Hausen. We hope you enjoyed the anamnesis conversation with Mr. and Mrs. Hausen. Please provide us with honest feedback so we can further develop this training tool in medical anamnesis conversations. Completing the survey will take approximately 10 minutes. All answers are collected anonymously and will be used for statistical purposes and scientific publications.  Many thanks for your support! |
| --- |
| 1. Question: Which discipline or study program do you belong to?  Answers:  • Nutrition Sciences  • Dietetics  • Logopedics  • Ergotherapy  • Physical Therapy  • Nursing  • Other: _____ |
| 2. Question: Do you already have practical experience with anamnesis conversations?  Answers:  • Yes  • No |
| 3. Question: How realistic was the virtual patient regarding his speech behavior and his verbal responses? [Linguistic behavior]  Answer range: Very realistic and natural * * * * Unnatural and/or not authentic |
| 4. Question: How understandable and clear were the statements of the virtual patient? [Understandability]  Answer range: Misunderstandable * * * * Understandable and clear |
| 5. Question: How well could the virtual patient respond to questions and provide information? [Responsiveness]  Answer range: Very poorly * * * * Very well |
| 6. Question: How broad and informative were the contents of the virtual patient? [Diversity of content (depth, information content)]  Answer range: Very extensive and informative * * * * Limited and insufficient |
| 7. Question: Assess the following statements for professional and content-related precision:  Answers:  • The clinical information was correctly reproduced by the virtual patient.  • The descriptions of the virtual patient were consistent with the corresponding disease pattern.  • The virtual patient used medical knowledge correctly.  • The virtual patient was able to provide sufficient information about his symptoms and medical history.  • The virtual patient provided the appropriate relevant medical backgrounds to the related questions.  Rate: excellent - Good - partly - Poor - Very poor |
| 8. Question: How precise were the patient's statements regarding the disease pattern? [Precision of the expressions regarding the disease]  Answer range: Imprecise or not relevant to the disease pattern * * * * Very precise and relevant to the disease pattern |
| 9. Question: How precise were the patient's statements regarding a typical patient? [Precision of expressions regarding a typical patient]  Answer range: Very precise and realistic for a patient * * * * Imprecise or not realistic for a patient |
| 10. Question: How close was this virtual anamnesis conversation to a real anamnesis conversation regarding...?  Answers:  • Articulation  • Emotional expressiveness  • Openness and honesty  • Understanding  • Initiative  • Reaction to complexity  Rate: Very close - fairly close - Neutral – some differences - unrealistic |
| 11. Question: How did you behave towards the virtual patient?  Answers:  • Respectful  • Empathic  • Unnatural  • Discriminatory  • More professional than in a real situation  Rate: fully agree – agree – partly – disagree – do not agree at all |
| 12. Question: What did you like the most about the conversation experience with the virtual patient?  Open Question |
| 13. Question: What did you like the least about the conversation experience with the virtual patient?  Open Question |
| 14. Question: In your opinion, what value do virtual anamnesis conversations add to the education in health professions?  Answer range: None * * * * Very high value |
| 15. Question: Which method for practicing anamnesis conversations in education would you like to try? (Multiple responses possible)  Answers:  • Real patient  • Actors  • Avatar  • Voice-based chat bots  • Chatbot (as in this study) |
| 16. Question: What are the advantages, in your opinion, of training anamnesis conversations with a chatbot?  Open question |
| 17. What are the disadvantages, in your opinion, of training anamnesis conversations with a chatbot?  Open question |
| 18. Question: Any other aspects/feedback you have regarding the virtual patient and the anamnesis conversation.  Open question |
